# Tailored N-Containing Carbons as Catalyst Supports in Alcohol Oxidation

**DOI:** 10.3390/ma9020114

**Published:** 2016-02-17

**Authors:** Sebastiano Campisi, Stefania Marzorati, Paolo Spontoni, Carine E. Chan-Thaw, Mariangela Longhi, Alberto Villa, Laura Prati

**Affiliations:** Dipartimento di Chimica, Università degli Studi di Milano, via C. Golgi 19, 20133 Milano, Italy; sebastiano.campisi@unimi.it (S.C.); stefania.marzorati@unimi.it (S.M.); paolo.spontoni@unimi.it (P.S.); carine.chanthaw@unimi.it (C.E.C.-T.); Mariangela.Longhi@unimi.it (M.L.); alberto.villa@unimi.it (A.V.)

**Keywords:** N-containing carbon, alcohol oxidation, Pd catalyzed oxidation

## Abstract

The introduction of N-containing functionalities in carbon-based materials is brought to stable and highly active metal-supported catalysts. However, up to now, the role of the amount and the nature of N-groups have not been completely clear. This study aims to clarify these aspects by preparing tailored N-containing carbons where different N-groups are introduced during the synthesis of the carbon material. These materials were used as the support for Pd nanoparticles. Testing these catalysts in alcohol oxidations and comparing the results with those obtained using Pd nanoparticles supported on different N-containing supports allowed us to obtain insight into the role of the different N-containing groups. In the cinnamyl alcohol oxidation, pyridine-like groups seem to favor both activity and selectivity toward cinnamaldehyde.

## 1. Introduction

**Figure 1:**
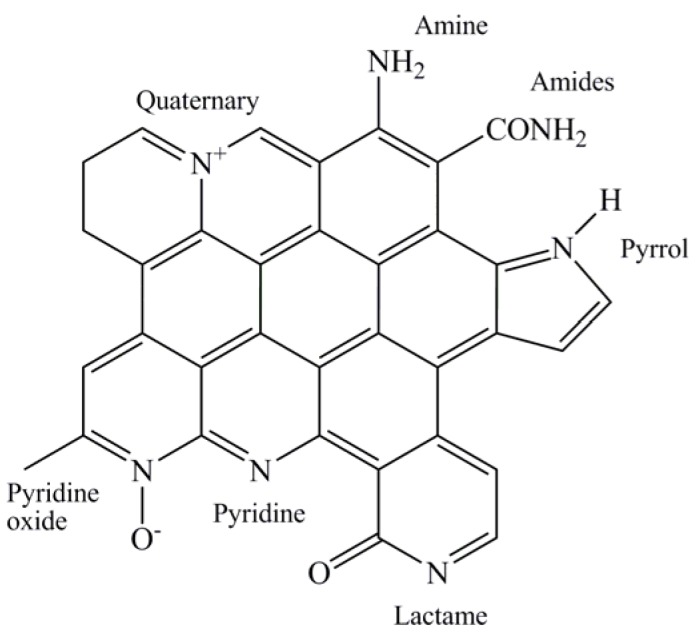
Different N-containing groups possibly present in carbons.

Carbons can be considered one of the most used classes of materials employed in heterogeneous catalysis [1,2,3,4]. Initially, their success was primarily due to their low cost and relatively good resistance to both acid and basic environments. An advantage more recently discovered is their flexibility in the modification of the surface, which can be tuned by introducing different heteroatoms using post-synthesis procedures. In particular, the use of N-modified carbons has found large application from the discovery that they can be usefully employed as a support for the cathode oxygen reduction reaction (ORR) catalyst in fuel cells [5]. N-doped carbons appear to commonly also be used in catalysis. In particular, thermal treatment of carbons in the presence of NH_3_ provides several types of N-containing functionalities within the carbon structure [6,7,8] whereas a higher control in N-functionalities can be obtained by using N-containing carbon precursors such as aromatic nitriles [9,10,11]. The former materials contain different types of N-functionalities, namely pyridine-like, amine/amide, pyrrol/pyridone, ammonium, and N-oxide groups, the ratio of which depends on the temperature of the thermal treatment with NH_3_ [7] (Figure 1). The latter materials, on the contrary, contain mainly two species: pyridine-like and pyrrol/pyridone [10].

When supporting Pd metal nanoparticles on all these materials, it clearly appears that the presence of N-groups enhanced the metallic dispersion, thus also enhancing catalytic activity as experienced in the liquid phase oxidation of glycerol [9] or benzyl alcohols [11]. Apparently, a correlation between the amount of N-content and the beneficial effect in promoting the catalytic activity was also found [12], as was a correlation between the location of the N-groups and the catalyst activity [13]. It was also revealed that, to assist a beneficial catalytic effect, the N atoms have to be within the carbon structure, acting most likely as anchoring centers for the metal nanoparticle.

However, X-ray photoelectron spectroscopy (XPS) studies showed relevant differences in the nature of the N-groups present on the different catalysts, the effect of which on the catalytic activity deserves further investigation to be clarified.

The present study aims to fill in this void by exploring the use of tailored N-doped carbons as the support for Pd nanoparticles in the liquid phase oxidation of alcohols. The use of a guanidine/glucose mixture as source of N and C, respectively, allows the N-doped carbon to contain a controlled amount of N and specific N-groups.

## 2. Results

### 2.1. N-Carbon Synthesis and Characterization

**Figure 2:**
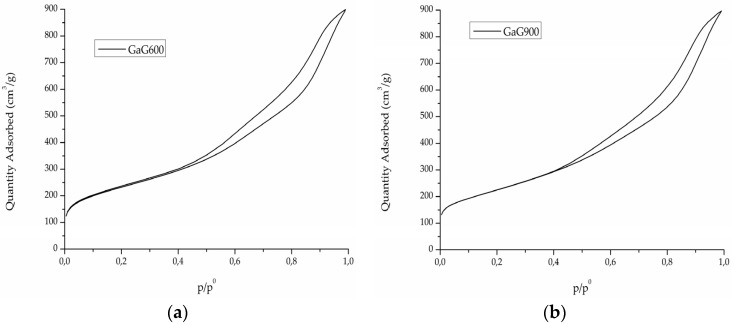
Isotherms of (**a**) GaG600 and (**b**) GaG900.

Carbon synthesis has been performed as reported in [14] using a hard templating technique. The as-synthesized sample labeled GaG600 was calcined at 900 °C, producing a new sample labeled GaG900. Isotherms of N_2_ adsorption for the two carbons are reported in Figure 2 ((a) GaG600; (b) GaG 900).

**Figure 3:**
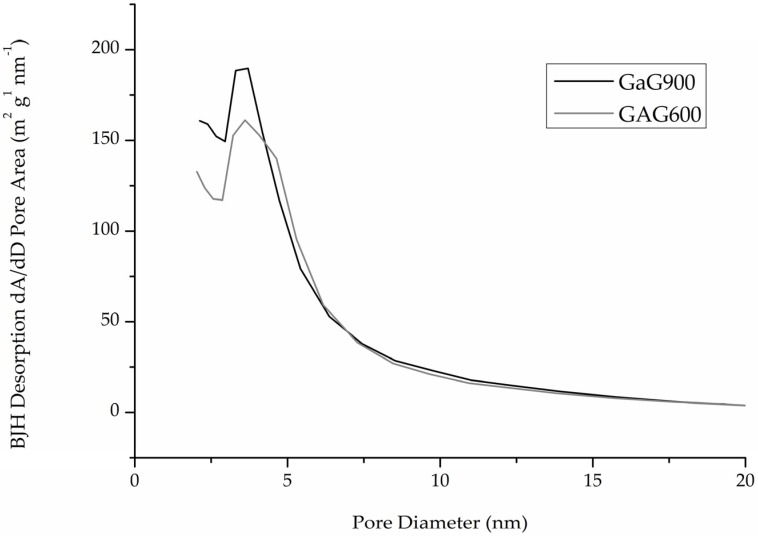
Porosity distribution (area-desorption part of the isotherm).

The porosity distribution of the two samples (Figure 3) did not show any relevant differences.

A slight decrease in the specific surface area from 826 m^2^/g to 793 m^2^/g was observed during calcination at the slight expense of microporosity, which was reduced from 12.2% in GaG600 to 8.5% in GaG900 (Table 1), even though the difference could be considered insignificant both in terms of porosity (Figure 3) and surface area (Table 1).

More significantly, the high temperature treatment also decreased the N content, as highlighted by elemental analyses (Table 2). GaG600 shows 15.5 wt % N instead of the 5.52 wt % found in GaG900. Is it of note that the H content also decreased after calcination, meaning that further mineralization occurs or that the N-groups eliminated also contain H.

**Figure 4:**
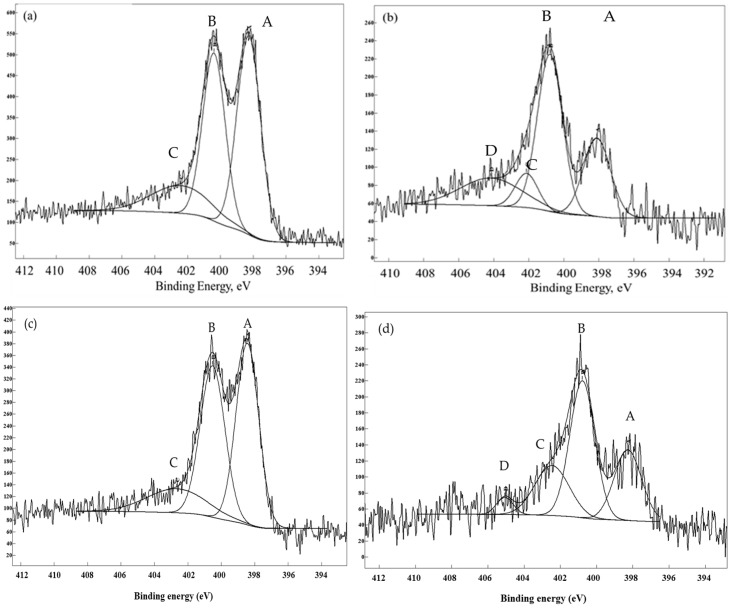
N1s analyses for GaG600 (**a**), GaG900 (**b**), Pd/GaG600 (**c**), and Pd/GaG900 (**d**), showing the presence of pyridinic (A), pyrrolic (B), pyridine oxide (C) and nitro-type nitrogen (D).

XPS analyses were performed in order to investigate the nature of the N-groups (Figure 4). Following the procedure from the literature [16,17,18] we established that the two main groups present in GaG600 are the pyridine- (398.4 eV) and pyrrole/pyridone-like groups (399.7 eV). A minor contribution was provided by pyridin oxide (403.2 eV). The high temperature treatment decreases the pyridine-like groups in accordance with what is reported in the literature: a decrease of pyridine in favor of pyrrole/pyridone occurs for high temperature treatments [7] (Figure 4a,b). GaG900 also presents nitro-type nitrogen (404.2 eV).

The XPS survey analyses (Table 3) highlighted a slight decrease in the oxygen content and pointed out that pyrrole-like groups are most probably the groups preferably formed. The overall composition at the surface is consistent with elemental analyses.

As benchmarks, we also used an active carbon treated with HNO_3_ and NH_3_ (N-AC) and a covalent triazine framework which used dicianopyridine (CTF_DCP_) prepared as reported in [9,10,11] as a starting material. The relevant characteristics of these two supports are reported in Table 3. In particular, CTF_DCP_ presented a similar N-content as GaG600 whereas N-AC showed a chemical composition similar to GaG900. CTF and N-AC, differently from GaGs, can be classified as microporous materials.

### 2.2. Pd Catalyst Preparation and Catalytic Test

**Figure 5:**
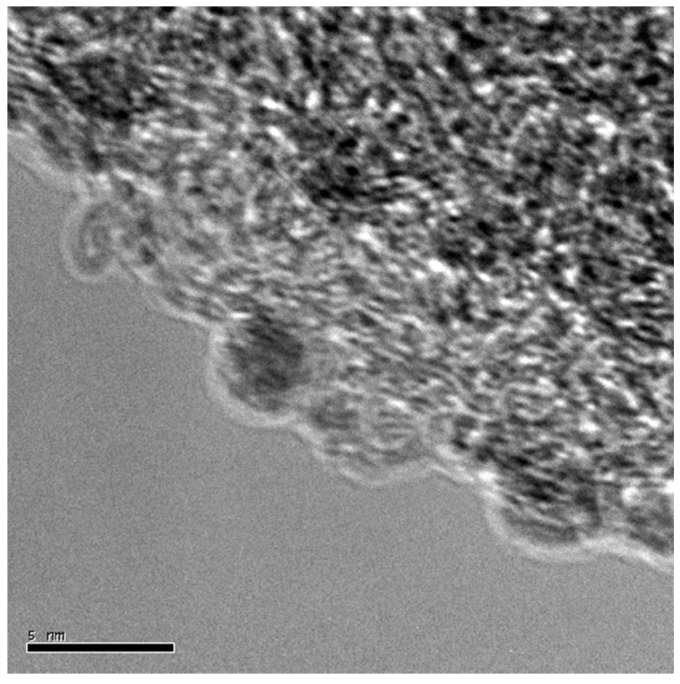
TEM representative image of Pd/GAG600.

GaG600 and GaG900 have been impregnated with a preformed Pd sol obtained by reducing Pd(II) salt with NaBH_4_ in the presence of polyvinylalcohol (PVA) as the protecting agent, following the procedure reported in [19]. The loading of the metal was in both cases 1 wt % and the obtained mean particle size was about 3 nm in both cases (Table 4). TEM images (Figure 5) confirmed a good metal dispersion in both cases.

The characterization of the catalysts in terms of specific surface area, pore distribution and elemental analysis did not vary appreciably from the bare supports most likely because of the low loading of the metal. The N1s region of the XPS spectra changed only in terms of overall intensity but not in terms of the internal intensity ratio, meaning that both types of groups are effective in coordinating Pd (Figure 4c,d, Table 4). However, the Pd atomic ratio at the surface appeared very different in the two samples (2.5 at% for GaG600 and 0.8 at% near the nominal value for GaG900) despite the similar mean size (Table 4). Supporting the same sol on CTF (covalent triazine framework) and N-AC (N-containing active carbon) with the same loading as for the GaGs, we obtained a similar particle size and atomic % (at%) for Pd by XPS similar to the nominal value in the case of Pd on GaG900 (Table 4). However, in the case of the CTF support, we observed a variation of the ratio between pyridine and pyrrolic-like groups after the immobilization of the metallic sol with a neat increase of pyridine groups (from 44:41 to 88:10) (Table 3 and Table 4), which is different from GaGs and N-AC. Concerning the Pd oxidation state, XPS revealed a 20%–30% presence of oxidized Pd in both samples, which is consistent with the exposure to air of the samples. The oxidation state could be relevant from a catalytic point of view as reported in [20]. Moreover, the use of preformed Pd nanoparticles excludes the presence of atomically dispersed Pd ions as in the case of impregnation [21].

We tested all the catalysts in alcohol oxidation using cinnamyl alcohol as the substrate in order to highlight the possible different roles of N-groups. Cinnamyl alcohol was oxidized with O_2_ in the presence of the catalyst (1:3000 Pd:alcohol) using p-xylene as the solvent (Table 5).

Pd/GaG600 and Pd/GaG900 showed a completely different catalytic activity, with Pd/GaG600 being the most active and selective (Table 5). Interestingly, Pd/CTF presented a higher activity than Pd/GaG600 whereas Pd/N-AC activity had results very similar to Pd/GaG900. Note that Pd/AC (the bare AC without N-groups) under the same conditions was only slightly less active than Pd on N-AC or GaG900.

## 3. Discussion

Two parent N-containing samples have been synthesized in order to determine the influence of the N-group’s nature in Pd-catalyzed alcohol oxidation. In recent studies, we experienced a general beneficial catalytic effect in modifying the carbon structure with N functionalities but no indication on the role of the single group has been revealed. The use of two N-containing carbons where one (GaG900) is derived from the other (GaG600) allowed us to avoid any interference of different structures. Indeed, the two samples showed similar surface area, closed mesoporous structure and similar types of N functionalities, even in a different ratio. Pd/GaG600 presents a surface (XPS) N-content of 11.3 at% more or less equally distributed between pyridine and pyrrole-like groups whereas in Pd/GaG900 the N-content is only 3.9 at% with a major contribution of pyrrole-like groups. The Pd nanoparticles showed a similar Pd mean size (3 nm) with similar content of oxidized palladium, but in GaG600 XPS data established a 2.5 at% of Pd at the surface instead of 0.8 at% in the case of GaG900. As the two samples show a comparable mesoporosity (Figure 3), we excluded any difference in terms of diffusion during the immobilization of the sol. Moreover, the effectiveness of the N-groups in coordinating Pd appears similar, as the internal ratio of the intensity of the peaks in XPS spectra remained similar before and after the Pd deposition. Therefore, we concluded that the difference in Pd at the surface of the two catalysts is due to the higher amount of N-groups in GaG600 than in GaG900. This would provide a more rapid anchoring of the Pd nanoparticle with a consequent higher deposition on the outer surface of GaG600. In contrast, a lower number of N-groups allows a more homogeneous distribution of Pd nanoparticles into the bulk of the material in the case of GaG900. This trend is also confirmed in the case of CTF, where the high N content fixes the Pd nanoparticle preferentially on the outer surface (Pd 4 at%, Table 4). However, in this latter case we observed a strong decrease of the pyrrolic group at the surface with respect to the bare support (pyridine/pyrrolic groups from 44:41 to 88:10 before and after Pd deposition, Table 4).

Considering the catalytic activity (Table 5), it clearly appears that Pd supported on GaG900 is definitely less active than on GaG600. However, due to the strongly different Pd exposition (revealed by XPS), we were not able to conclude if the differences of N-groups on the supports were relevant or not. Therefore, we considered the activity of Pd supported on CTF and N-AC, as they have a similar elemental composition as GaG600 and 900, respectively (Table 3). CTF, after immobilization of the Pd sol, presents an atomic ratio of C–N–Pd at the surface (XPS) of 86.4–9.4–4.1 and a relative ratio of pyridinic/pyrrolic groups of 88:10 (Table 4). N-AC, on the other hand, presents an atomic ratio of C–N–Pd at the surface (XPS) of 98:1:0.9 and a relative ratio of pyridinic/pyrrolic groups of 43:57 (Table 4), thus it has a prevalence of pyrrolic groups as in the case of Pd/GaG900 (30:70).

As reported in Table 5, we observed a reactivity in the order of Pd/CTF > Pd/GaG600 > Pd/N-AC > Pd/GaG900 > Pd/AC. All the catalysts have a similar Pd mean size and the activity follows, though not linearly, the pyridine-like group presence (88:10 CTF; 57:43 GaG600; 43:57 N-AC; 25:46 GaG900). The Pd exposition decreases in the order of 4.1 at% for CTF, 2.5 at% for GaG600, 0.8 at% for GaG900, 0.9 at% for N-AC. Therefore, Pd on GaG900 on the basis of Pd exposition should show the same activity as Pd on N-AC. Moreover, the surface N-content for Pd/N-AC, even if slightly higher in the bulk, resulted in less than Pd/GaG900. As we demonstrated that the catalytic activity increases by increasing the surface N amount [13], we conclude that the higher activity of Pd/N-AC compared to that of Pd/GaG900 is probably due to the higher content of the pyridinic groups. The higher activity of Pd/CTF can thus be explained by the highest content of N-pyridinic groups, even though we cannot exclude a contribution of the highest Pd exposition.

## 4. Materials and Methods

### 4.1. Materials

All chemicals and reagents were used as received without further purification. *D*-glucose, guanidine acetate, glacial acetic acid were purchased from Sigma Aldrich (Milan, Italy). Silica (70–230 mesh) was from Merck (Milan, Italy).

### 4.2. Methods

#### 4.2.1. Carbon Syntheses

Functionalized carbons synthesis was reported in Reference [14]. A solution containing glucose (Glu) 1.68 mol dm^−3^, guanidine acetate (GA) and glacial acetic acid in a Glu:GA:Acetic Acid = 1:1:3 molar ratio was prepared. This solution was soaked in a silica powder to obtain a gel. This gel was loaded in a quartz reactor, degassed with N_2_ (100 cm^3^·min^−1^) for about 5 min and inserted in a preheated vertical oven at *T* = 600 °C for 1 h while continuing N_2_ purging (100 cm^3^·min^−1^). Then the tube was rapidly quenched in air. Silica was removed in 3 M boiling NaOH followed by repeated washing/filtering (MilliQ water, 0.45 μm Durapore filters) of carbon products until water conductivity was lower than 4 μS. Products were dried in nitrogen (100 °C, 24 h) and finally ground in an agate mortar (in the following this sample will be labeled GaG600).

GaG600 was also heat-activated in a second step at *T* = 900 °C under constant N_2_ flow (100 cm^3^·min^−1^) in the following conditions: 30 min room temperature purging, ramping at 6 °C·min^−1^ and 3 h standing at *T* = 900 °C, fast-quenching to room temperature (in the following this sample will be labeled GaG900).

#### 4.2.2. Pd on Carbon Catalysts

GaG600 and GaG900 have been used as support for Pd metallic sol obtained as follows: Pd sol preparation: Na_2_PdCl_4_·2H_2_O (0.043 mmol) salt and freshly prepared 1 wt % PVA solution were added to 130 mL of H_2_O (Pd/PVA ratio 1:1 *wt*/*wt*). After 3 min, NaBH4 0.1 M solution (Pd/NaBH_4_ 1/8 mol/mol) was added to the yellow-brown solution under vigorous magnetic stirring. The brown Pd(0) sol was immediately formed. An UV-visible spectrum of the palladium sol was recorded for ensuring the complete reduction of Pd (II). Within few minutes from its generation, the suspension was acidified at pH 2 by sulphuric acid and the support was added under vigorous stirring. The catalyst was filtered and washed for several times with distilled water. The samples were dried at 80 °C for 2 h. The amount of the support was calculated to obtain a final metal loading of 1 wt %.

The catalyst obtained were labeled as Pd/GaG600 and Pd/GaG900.

#### 4.2.3. Catalytic Test

The reactions were carried out in a thermostatted glass reactor (30 mL) agitated with an electronically controlled magnetic stirrer connected to a large reservoir (5000 mL) containing oxygen at 2 atm. The oxygen uptake was followed by a mass flow controller connected to a PC through an A/D board. The oxidation experiments were carried out in xylene (1.25 M substrate, substrate/Pd = 3000 (mol/mol), 80 °C, *p*_O2_ = 2 atm). The reaction was monitored by analyzing periodically withdrawn samples. Mass balances, in the analysis, were always 98% ± 3%. Analyses were performed using a HP 7820A gas chromatograph equipped with a capillary column HP-5 30 m × 0.32 mm, 0.25 μm Film, by Agilent Technologies(Milan, Italy). Authentic samples were analyzed to determine separation times. Quantitative analyses with external standard method (*n*-octanol) was used.

### 4.3. Characterization

Surface area and porosity distribution were determined by low temperature (*T* = −196 °C) N_2_ adsorption using an Tristar II 3020 Micromeritics apparatus (Milan, Italy). Before measurement, samples were outgassed at *T* = 150 °C for 4 h in a nitrogen flux. Surface area and porosity distribution were calculated from nitrogen isotherms by Brunauer, Emmett, Teller and Barrett, Joyner, Halenda (B.E.T. and B.J.H.) theories using the instrumental software (Version 1.03).

ICP analyses have been performed on filtered solution and revealed a quantitative adsorption of the metals (1 wt %).

Elemental analyses have been performed on a PerkinElmer 2400 Series II, CHNS/O Elemental Analyzer (Milan, Italy).

## Figures and Tables

**Table 1:** Specific surface area and other characteristics of N-carbons.

Measure	GaG600	GaG900
Specific Surface Area (m^2^·g^−1^)	826 ± 5	793 ± 5
Micropores (m^2^·g^−1^)	101 (12.2%)	67 (8.5%)
Qm/ mmol (g^−1^) ^a^	8.5	8.1

^a^ Qm = Monolayer Capacity, calculated from BET equation [15].

**Table 2:** Elemental analyses (C, H, N).

Supports	C (wt %)	H (wt %)	N (wt %)
GaG600	74.64	2.22	15.5
GaG900	88.18	0.77	5.52

**Table 3:** Characteristics of N-containing supports.

Catalyst	XPS					Elemental Analysis
	N1S (%)				Atomic Ratio	C–H–N (wt %)
	Pyridinic	Pyrrolic	Pyridin Oxide	NO	C–O–N	
CTF_DCP_	44.3	41.5	10.3	3.9	77.8–8.4–13.8	64.0–1.0–18.0
N-AC	42.0	58.0	–	–	85.4–11.3–1.5	86.5–0.9–1.7
AC	–	–	–	–	–	85.1–1.1–0.1
GAG600	46.2	37.4	16.4	–	83.5–3.0–13.5	74.6–2.2–15.6
GAG900	24.6	45.6	8.7	21.1	92.3–2.4–5.3	88.2–0.8–5.5
**BET**						**Reference**
**Pore Size (nm)**		**Surface Area (m^2^/g)**				
not determined		1738				[9,10]
2.4		1048				[13]
2.1		1100				[13]
7.3		826				This work
8.0		793				This work

**Table 4:** Characteristics of Pd catalysts.

Catalyst	TEM	XPS					Pd0/Pd^δ+^	Reference
	Pd Size (nm)	N1S (%)				Atomic Ratio		
		Pyridinic	Pyrrolic	Pyridin Oxide	NO	C–N–Pd		
Pd/ CTF_DCP_	3.1 ± 0.9	88.4	9.9	–	1.7	86.4–9.4–4.1	76.4/23.6	[9,10]
Pd/N-AC	3.5 ± 0.9	43.3	56.7	–	–	98.1–1.0–0.9	64.4/35.6	[13]
Pd/AC	3.9 ± 1.2	–	–	–	–	98.6–0.0–1.4	65.9/34.1	[13]
Pd GAG600	3.5 ± 0.7	44.4	40.3	15.3	–	75.1–11.2–2.5	69.8/30.2	This work
Pd GAG900	3.7 ± 1.2	25.8	46.5	22.4	5.3	88.8–3.9–0.8	66.1/33.9	This work

**Table 5:** Alcohol oxidation ^a^.

Catalyst (1 wt % Pd)	Activity ^b^	Selectivity at 50% Conversion		
		Cinnamyl Aldehyde	3-Phenyl-1-Propanol	Styrene
Pd/CTF	253	79	20	1
Pd/N-AC	86	78	22	–
Pd/AC	54	67	30	3
Pd GAG600	209	82	18	–
Pd GAG900	78	78	22	–

**^a^** Reaction conditions: Alcohol 1.25 M in *p*-Xylene, 80 °C, *p*_O2_ 2 atm, metal/alcohol 1/3000 mol/mol; **^b^** activity measured as converted mol_alcohol_ mol_Pd_^−1^·h^−1^. The reactions were followed for 6 h and selectivity reported at 50% conversion.

## References

[1] Rodríguez-Reinoso F. (1998). The role of carbon materials in heterogeneous catalysis. Carbon.

[2] Prati L., Villa A., Lupini A.R., Veith G.M. (2012). Gold on carbon: one billion catalysts under a single label. Phys. Chem. Chem. Phys..

[3] Serp P., Corrias M., Kalck P. (2003). Carbon nanotubes and nanofibers in catalysis. Appl. Catal. A General.

[4] Tessonnier J.P., Ersen O., Weinberg G., Pham-Huu C., Su D.S., Schlogl R. (2009). Selective deposition of metal nanoparticles inside or outside multiwalled carbon nanotubes. ACS Nano.

[5] Feng L., Yang L., Huang Z., Luo J., Li M., Wang D., Chen Y. (2013). Enhancing Electrocatalytic Oxygen Reduction on Nitrogen-Doped Graphene by Active Sites Implantation. Sci. Rep..

[6] Arrigo R., Wrabetz S., Schuster M.E., Wang D., Villa A., Rosenthal D., Girsgdies F., Weinberg G., Prati L., Schlögl R. (2012). Tailoring the morphology of Pd nanoparticles on CNTs by nitrogen and oxygen functionalization. Phys. Chem. Chem. Phys..

[7] Arrigo R., Hävecker M., Wrabetz S., Blume R., Lerch M., McGregor J., Parrott E.P.J., Zeitler J.A., Gladden L.F., Knop-Gericke A. (2010). Tuning the acid/base properties of nanocarbons by functionalization via amination. J. Am. Chem. Soc..

[8] Prati L., Villa A., Chan-Thaw C.E., Arrigo R., Wang D., Su D.S. (2011). Gold catalyzed liquid phase oxidation of alcohol: the issue of selectivity. Faraday Discuss..

[9] Chan-Thaw C.E., Villa A., Katekomol P., Su D., Thomas A., Prati L. (2010). Covalent triazine framework as catalytic support for liquid phase reaction. Nano Lett..

[10] Chan-Thaw C.E., Villa A., Veith G.M., Kailasam K., Adamczyk L.A., Unocic R.R., Prati L., Thomas A. (2012). Influence of Periodic Nitrogen Functionality on the Selective Oxidation of Alcohols. Chem. Asian J..

[11] Chan-Thaw C.E., Villa A., Prati L., Thomas A. (2011). Triazine-Based Polymers as Nanostructured Supports for the Liquid-Phase Oxidation of Alcohols. Chem. Eur. J..

[12] Chan-Thaw C.E., Villa A., Wang D., Santo V.D., Orbelli Biroli A., Veith G.M., Thomas A., Prati L. (2015). PdH*_x_* Entrapped in a Covalent Triazine Framework Modulates Selectivity in Glycerol Oxidation. ChemCatChem.

[13] Chan-Thaw C.E., Villa A., Veith G.M., Prati L. (2015). Identifying the Role of N-Heteroatom Location in the Activity of Metal Catalysts for Alcohol Oxidation. ChemCatChem.

[14] Galbiati I., Bianchi C.L., Longhi M., Formaro L., Carrà A. (2010). Iron and copper containing oxygen reduction catalysts from templated glucose–histidine. Fuel Cells.

[15] Sing K.S.W., Everett D.H., Haul R.A.W., Moscou L., Pierotti R.A., Rouquerol J., Siemieniewska T. (1985). Reporting physisorption data for gas/solid systems with Special Reference to the Determination of Surface Area and Porosity. Pure Appl. Chem..

[16] Moulder J.F., Stickle W.F., Sobol P.E., Bomben K.D. (1992). Handbook of Xray Photoelectron Spectroscopy.

[17] Boudou J.P., Chehimi M., Broniek E., Siemieniewska T., Bimer J. (2003). Adsorption of H_2_S or SO_2_ on an activated carbon cloth modified by ammonia treatment. Carbon.

[18] Kapteijn F., Moulijn J.A., Matzner S., Boehm H.P. (1999). The development of nitrogen functionality in model chars during gasification in CO_2_ and O_2_. Carbon.

[19] Wang D., Villa A., Porta F., Su D., Prati L. (2006). Single-phase bimetallic system for the selective oxidation of glycerol to glycerate. Chem. Commun..

[20] Grunwaldt J.-D., Caravati M., Baiker A. (2006). Oxidic or Metallic Palladium: Which Is the Active Phase in Pd-Catalyzed Aerobic Alcohol Oxidation?. J. Phys. Chem. B.

[21] Vilé G., Albani D., Nachtegaal M., Chen Z., Dontsova D., Antonietti M., López N., Pérez-Ramírez J. (2015). A Stable Single-Site Palladium Catalyst for Hydrogenations. Angew. Chem. Int. Ed..

